# n-Butyl Benzyl Phthalate Exposure Promotes Lesion Survival in a Murine Endometriosis Model

**DOI:** 10.3390/ijerph18073640

**Published:** 2021-03-31

**Authors:** Pooja Sharma, Jo-Yu Lynn Lee, Eing-Mei Tsai, Yu Chang, Jau-Ling Suen

**Affiliations:** 1 Graduate Institute of Medicine, College of Medicine, Kaohsiung Medical University, Kaohsiung 80708, Taiwan; poojasharma10113@gmail.com (P.S.); lynnjylee@gmail.com (J.-Y.L.L.); tsaieing@yahoo.com (E.-M.T.); 2 Department of Obstetrics and Gynecology, Kaohsiung Medical University Hospital, Kaohsiung 80756, Taiwan; 3 Department of Obstetrics and Gynecology, E-Da Hospital, I-Shou University, Kaohsiung 82445, Taiwan; changyu0510@gmail.com; 4 Research Center for Environmental Medicine, Kaohsiung Medical University, Kaohsiung 80708, Taiwan; 5 Department of Medical Research, Kaohsiung Medical University Hospital, Kaohsiung 80756, Taiwan

**Keywords:** n-Butyl benzyl phthalate, CD44, dendritic cell, endometriosis

## Abstract

Endometriosis is an inflammatory and estrogen-dependent gynecological disease associated with exposure to environmental endocrine disruptors. n-Butyl benzyl phthalate (BBP), a ubiquitous plasticizer, has weak estrogenic activity, and exposure to BBP is associated with endometriosis. We aimed to elucidate the immunomodulatory effect of BBP on endometriosis development. We previously established a surgery-induced endometriosis-like murine model. In the present study, we exposed those mice to BBP 10 days prior to surgery and 4 weeks after surgery at physiologically relevant doses to mimic human exposure. Chronic exposure to BBP did not promote the growth of endometriotic lesions; however, the lesion survival rate in BBP-treated mice did increase significantly compared with control mice. Multiparametric flow cytometry showed that BBP exposure did not affect the homeostasis of infiltrated immune subsets in lesions but did enhance CD44 (adhesion marker) expression on plasmacytoid dendritic cells (pDCs). Blocking CD44 interactions locally inhibited endometriotic lesion growth. Immunofluorescence results further confirmed that CD44 blocking inhibited pDC infiltration and reduced the frequency of CD44^+^ pDCs in endometriotic tissues. BBP also disrupted the estrus cycle in these mice. This study suggests that chronic exposure to low-dose BBP may promote survival of endometriotic tissue through CD44-expressing pDCs.

## 1. Introduction

Endometriosis is an inflammatory and gynecological disorder defined as the presence of endometrium-like tissue outside the uterine cavity. The prevalence of endometriosis is about 5–10% among women of reproductive age and is associated with pelvic pain, infertility, dyspareunia, and dysmenorrhea [1]. The etiology of endometriosis is still unclear, although several theories have been proposed regarding its pathogenesis, including coelomic metaplasia; Müllerian remnants; and, the most widely accepted theory, retrograde menstruation (attachment and adhesion of endometrial cells to the peritoneal cavity) [2]. Whereas retrograde menstruation and the presence of viable endometrial cells in peritoneal fluid occur in 90% of women, endometriosis occurs when those endometrial cells escape immune clearance, become implanted in and attach to the peritoneal epithelium, and grow and survive [3]. Endometriosis pathogenesis is facilitated by multiple factors including inflammation, genetic predisposition, estrogen dependence, and progesterone resistance [4].

Endometrial cells that form endometriotic lesions in the peritoneal cavity do so within a complex immune microenvironment that is dominated by endocrine, angiogenic, and inflammatory mediators. A variety of innate immune cells are involved in the development of endometriosis including neutrophils, monocytes, macrophages, natural killer cells, conventional dendritic cells (cDCs), and plasmacytoid dendritic cells (pDCs) [5,6,7]. pDCs are a well-known innate immune subset that maintains antiviral immunity by producing type I interferons and exhibits proinflammatory or tolerogenic properties depending on disease context [8]. In our previous study, using a surgically induced murine model, we showed that interleukin-10 (IL-10)-secreting pDCs are the major subset among infiltrated CD45^+^ cells in endometriotic lesions and that IL-10 from pDCs promotes endometriosis pathogenesis through angiogenesis in an IL-10R-dependent manner during the early stage of disease [6,7]. The detailed mechanisms of pDC involvement in endometriosis pathogenesis need further investigation.

It has been reported that the environmental endocrine disruptor (EED) is a critical risk factor for endometriosis, such as dioxin [9], heavy metals [10,11,12], and phthalates [13]. n-Butyl benzyl phthalate (BBP), a widely used plasticizer, is known to be an EED as it has weak estrogenic activity, and its exposure is associated with endometriosis. BBP is commonly found in medical products, lubricants, automotive trims, food packaging, and children’s toys [14]. BBP is released from discarded plastics into aqueous environments and may enter the food chain [15,16]. In vitro BBP has weak estrogenic activity (relative binding affinity is 0.02- to 0.01-fold for human recombinant estrogen receptor-α (ERα)) [17] and competes with 17β-estradiol (E2) for binding to the estrogen receptor (ER) [18]. BBP also induces ERα-mediated proliferation in the breast cancer cell line MCF-7 [19] and decreases the myogenic differentiation of endometrial mesenchymal stem/stromal cells through epigenetic regulation [20]. Upson et al. [13] demonstrated that urinary concentrations of MBzP (mono-n-benzyl phthalate), a metabolite of BBP, may be associated with an increased risk of endometriosis. An additional study found an etiological association between phthalate esters such as BBP and the occurrence of endometriosis [21].

To explore the causal relationship between BBP exposure and endometriosis pathogenesis, we used an established surgically induced endometriosis model in conjunction with exposure to BBP under relevant routes and levels to mimic human exposure. 

## 2. Materials and Methods

### 2.1. Mice and the Surgically Induced Endometriosis Model

The procedure was performed on C57BL/6 female mice at 4 weeks of age. The animals were obtained from the National Laboratory Animal Center (Taipei, Taiwan) and maintained at Kaohsiung Medical University in a specific pathogen-free environment. The mice were maintained on a 12 h dark/light cycle at 21 ± 2 °C and 30−35% relative humidity with access to food and water *ad libitum* in the animal facility. Each experiment included 4–6 mice in each group, and each experiment was independently repeated 2–4 times. The mice were orally fed BBP (Sigma-Aldrich, St. Louis, MO, USA) daily to mimic human exposure based on the human tolerable daily intake (TDI) dose of 0.5 mg/kg body weight (BW)/day, as determined by the European Food Safety Authority [22], or were fed a 3-fold TDI dose, that is, 1.5 mg/kg BW/day. Before endometriosis induction by surgery, animals were orally fed BBP for 10 days, with surgery for endometriosis induction occurring on Day 11. One day after surgical induction (Day 12), the mice were again exposed to BBP as described above for an additional 4 weeks (Supplementary Figure S1). Mice were orally fed with 0.1% DMSO in corn oil served as vehicle controls. Mice were orally fed with E2 (50 µg/kg BW/day; Sigma-Aldrich) or 0.1% DMSO for continuously 22 days without surgery for estrus cycle analysis.

The endometriosis model was established according to our previous study [7]. In brief, autologous uterine horns from the treated mice were punched to generate four identically sized round tissue samples (2 mm in diameter), and two tissues were then surgically sutured to left and two on the right side of the peritoneal wall for the formation of ectopic lesions. If all four sutured lesions from the left and right side were harvested from each surgically treated mouse at the end of the study, the lesion survival rate was defined as 100%.

In some experiments, blocking monoclonal antibody against CD44 and its corresponding isotype control (20 µg/mL, 20 µL/lesion; BioLegend, San Diego, CA, USA) were intradermally injected into the peritoneal wall under each transplanted tissue on the right side and left side, respectively, as shown in (Supplementary Figure S2A). Four weeks after surgery, the lesions were collected and weighed, and the lesion area was measured using free image analysis software (ImageJ Software 1.46r, NIH, Bethesda, MD, USA). The infiltrated immune cell subsets were analyzed using a multiparametric flow cytometer (LSR II, BD Biosciences, San Diego, CA, USA) and FlowJo software (version 10, Tree Star, Inc., Ashland, OR, USA).

### 2.2. Flow Cytometry for Analysis of Immune Cell Subsets

The pooled endometriotic lesions from each mouse were processed to become single-cell suspensions. The lesions were incubated in 0.05% trypsin, 0.1% collagenase D, 0.53 mmol/L EDTA, and 150 mg/mL DNase I for 40 min and were then mechanically disrupted with a gentle MACS dissociator (Miltenyi Biotec, Auburn, CA, USA) according to the manufacturer’s instructions. The lesion single-cell suspensions were stained with PerCP/Cy5.5-conjugated anti-CD45 (30-F11, BD Biosciences), APC-conjugated anti-CD11c (N418, eBioscience, San Diego, CA, USA), PE-conjugated anti-PDCA-1 (eBio927, eBioscience), PerCP/Cy5.5-conjugated anti-Ly6C (HK1.4, BioLegend), BV421-conjugated anti-F4/80 (T45-2342, BD Biosciences), FITC-conjugated anti-CD11b (M1/70, BioLegend), Alexa Fluor 700-conjugated anti-CD44 (IM7, BioLegend), PE-conjugated anti-CD45 (30-F11, BD Biosciences), and FITC-conjugated anti-ICAM-1 (YN1/1.7.4, BioLegend).

### 2.3. Immunofluorescence Staining

Tissue sections (5 μm thick) were prepared from frozen individual lesion samples and fixed with 4% paraformaldehyde, followed by incubation with primary antibodies rat anti-mouse PDCA-1 (1:100; BioLegend) and rabbit anti-mouse CD44 antibody (1:100; Abcam, Cambridge, UK) overnight at 4 °C. The sections were washed with PBS and then incubated with secondary antibodies Alexa Fluor 488-conjugated donkey anti-rat IgG (1:500; Invitrogen) and Alexa Fluor 568-conjugated goat anti-rabbit IgG (1:500; Invitrogen) for 1 h at room temperature. Finally, cell nuclei were counterstained with DAPI (1 μg/mL, Sigma-Aldrich, Darmstadt, Germany) for 5 min at room temperature. For negative control staining, sections were stained only with DAPI and secondary antibodies. The stained lesions were scanned with the TissueFAXS imaging system (Tissue Gnostics, Tarzana, CA, USA), and images were captured by confocal microscopy. An FV1000-HSD (OLYMPUS) microscope with UPLSAPO100× oil objective lens (zoom: ×4.5) was used, and the image was remodeled using Imaris software as a three-dimensional picture (Oxford Instruments, Zurich, Switzerland).

### 2.4. Vaginal Cytology

Vaginal lavage was performed early each morning to determine cell cytology. Cells were collected from the vaginal canal by inserting ~20 μL PBS with a micropipette. The solution was flushed twice and then was withdrawn and placed on a glass slide in a thin layer and allowed to air dry. The cells were stained with 0.1% crystal violet solution for 1 min. The slide was then washed with ddH_2_O, and the vaginal smear was covered with mounting medium and a coverslip. The slides were observed under a microscope. The ratio of cells present in the smear was used to determine the estrous cycle stage at the time of sample collection. Proestrus is defined by the presence of mostly nucleated epithelial cells having lightly stained cytoplasm with an oval nucleus. Estrus is defined by the presence of mainly densely packed cornified anucleated epithelial cells that are polygonal in shape. Metestrus is defined by the presence of predominately darkly stained small-sized neutrophils with some anucleated cornified epithelial cells. During diestrus, neutrophils still predominate, and some nucleated epithelial cells are present [23].

### 2.5. Statistical Analyses

Statistical comparisons of data among control and experimental groups were performed with the nonparametric Mann–Whitney U test. One-way ANOVA followed by Tukey’s multiple comparison test was used for analyzing differences among four groups. The comparison in number of mice with regular cycles was performed with the Mid-P exact test (OpenEpi, free and open-source software for epidemiological statistics; https://www.openepi.com/Menu/OE_Menu.htm, accessed on 22 November 2020). Values of *p* < 0.05 were considered significant. All statistical tests were performed with GraphPad Prism 5.0.

## 3. Results

### 3.1. Chronic Exposure to Low-Dose BBP Promotes Endometriotic Lesion Survival in a Surgically Induced Murine Model

**Figure 1:**
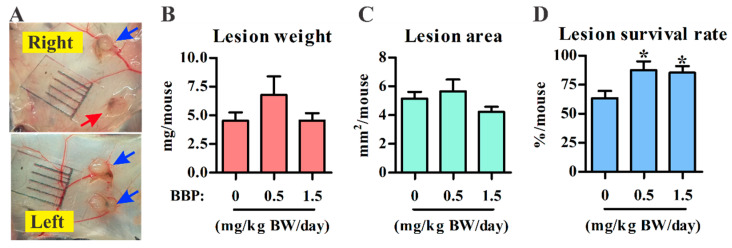
The effect of n-Butyl benzyl phthalate (BBP) on endometriosis development in a surgically induced model. Endometriotic lesions were harvested after 4 weeks from the surgically induced control mice (0 BBP) and from the two BBP treatment groups (0.5 and 1.5 mg BBP/kg BW/day). (**A**) The four lesions harvested from one representative control mouse. The growth lesions are identified with blue arrows, whereas the nongrowth lesion is indicated with a red arrow. (**B**,**C**) The mean weight (**B**) and mean area (**C**) of lesions pooled from each mouse are shown. (**D**) The lesion survival rate for each mouse was calculated from three independent experiments (control, *n* = 11; 0.5 mg BBP, *n* = 10; 1.5 mg BBP, *n* = 12). Results are shown as the mean ± SEM. * *p* < 0.05 vs. control with the Mann–Whitney U test.

To mimic human exposure to BBP via a relevant route and at an appropriate dose, the female mice were orally gavaged with corn oil (control group), 1× TDI BBP (0.5 mg/kg BW/day), or 3× TDI BBP (1.5 mg/kg BW/day). The BBP TDI dose is obviously higher than the estimated daily intake for Chinese women aged 18–59 years (median, 0.87 μg/kg BW/day, around 0.18% TDI) [24] or in the German population (min–max, 0.01–27.3 μg/kg BW/day, around 0.002–5.46% TDI) [25]; however, the doses used here are at least 1000-fold lower than those typically used in toxicological studies (>500 mg/kg BW/day) [26,27]. Therefore, both doses used in the present study can be considered low doses for BBP exposure. As shown in Figure 1A, one representative control mouse showed that four sutured tissue fragments resulted in the formation of three fluid-filled ectopic lesions on the peritoneal wall. The mean lesion weight and area in each mouse were not affected by chronic exposure to low-dose BBP (Figure 1B,C), but the lesion survival rate was significantly increased by BBP exposure in the surgically induced murine model (Figure 1D).

### 3.2. BBP-Mediated Lesion Survival Is Not Associated with Infiltration of Innate Immune Subsets in Lesions

**Figure 2:**
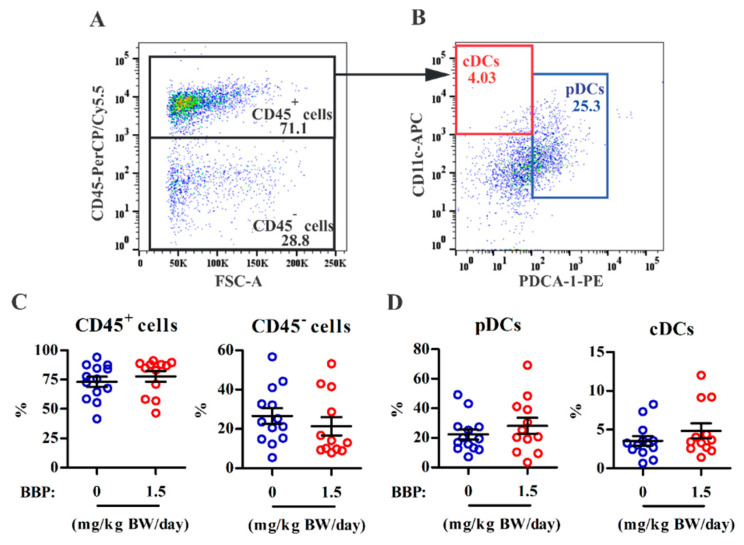
The effect of BBP on immune cell infiltration in endometriotic lesions. Endometriotic lesions were pooled from each single mouse for the analysis of immune cell subsets using multiparametric flow cytometry. (**A**) Representative dot plots are shown for gating for CD45^+^ (immune) cells and CD45^−^ (nonimmune) cells among viable cells and (**B**) gating for plasmacytoid dendritic cells (pDCs) (PDCA-1^+^CD11c^low^) and conventional dendritic cells (cDCs) (PDCA-1^−^CD11c^+^) among CD45^+^ cells in lesions. (**C**,**D**) The percentages of CD45^+^ cells and CD45^−^ cells (**C**) and pDCs and cDCs among the CD45^+^ cells (**D**) in pooled lesions from each mouse (*n* = 12 or 13 mice/group from four independent experiments). Each data point represents data from one mouse. The horizontal lines and error bars represent the mean and SEM, respectively, for each group.

As previous studies have demonstrated that pDCs [28], cDCs [29], monocytes, and macrophages [30] are associated with endometriosis pathogenesis, we next determined whether BBP exposure altered immune cell subset infiltration in endometriotic lesions. Multiparametric flow analysis showed that there was no difference in the percentages of CD45^+^ immune cells and CD45^−^ nonimmune cells between the control and 3× TDI BBP groups (Figure 2A–C). Among CD45^+^ immune cells, the pDC subset (CD11c^low^PDCA-1^+^; 22 ± 3%, mean ± SEM) had ~5-fold higher numbers than the cDC subset (CD11^+^PDCA-1^−^; 4 ± 0.6%) in the control group. BBP exposure did not alter the proportion of DC subsets in lesions (Figure 2D). In addition, the monocyte (CD11c^−^Ly6C^+^CD11b^+^F4/80^low^; 44 ± 4% in control) and macrophage (CD11c^+^Ly6C^−^CD11b^+^F4/80^high^; 1 ± 0.5% in control) subsets in lesions were not affected by BBP exposure (Supplementary Figure S3). These results suggest that BBP-mediated lesion survival cannot be attributed to the composition or proportion of innate immune subsets in endometriotic lesions.

### 3.3. BBP Exposure Enhances Adhesion Marker Expression on Infiltrating pDCs

**Figure 3:**
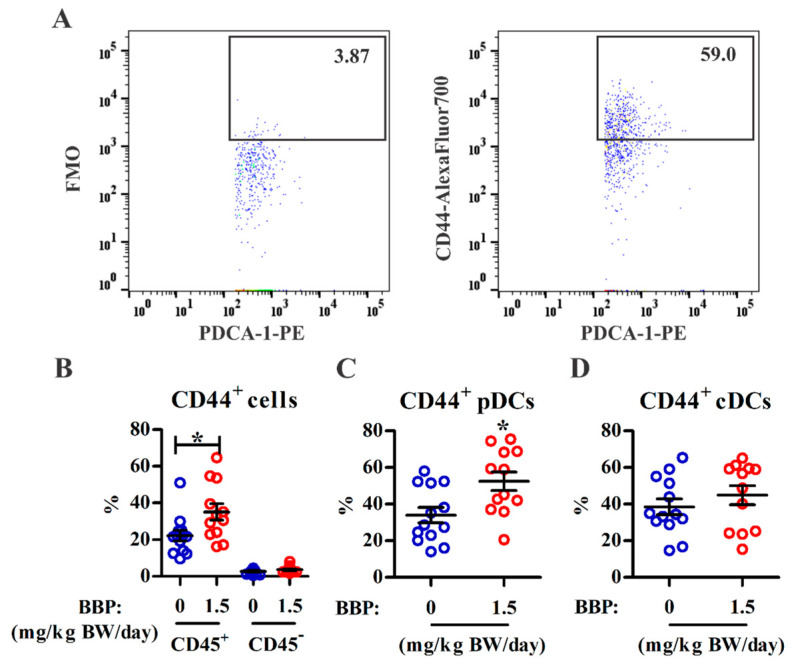
BBP exposure enhances CD44 expression in the pDC subset. Endometriotic lesions were pooled from each single mouse for analysis of CD44 expression among DC subsets. (**A**) Representative dot plots are shown for CD44 expression in pDCs from BBP-treated mice (1.5 mg/kg BW/day). The CD44^+^ cells were gated according to the fluorescence minus one (FMO) control. (**B**) Percentages of CD44^+^ cells among CD45^+^ immune and CD45^−^ nonimmune cells in lesions from control and 1.5 mg BBP groups. (**C**,**D**) Percentages of CD44^+^ cells among pDCs (**C**) and cDCs (**D**) from control and 1.5 mg BBP lesions (*n* = 12 or 13 mice/group from four independent experiments). Each data point represents data from one mouse. The black horizontal lines and error bars represent the mean and SEM for each group, respectively. * *p* < 0.05 vs. control based on the Mann–Whitney U test.

The expression of CD44 and ICAM-1 in ectopic endometrial cells may promote adhesion and enhance endometriosis pathogenesis [31]. We further analyzed the effect of BBP on ICAM-1 and CD44 expression in lesions using multiparametric flow cytometry. The percentage of CD44-expressing cells among the CD45^+^ immune cells was significantly increased in lesions from the BBP-treated mice (Figure 3A,B). CD45^−^ nonimmune cells did not express CD44 in lesions from either group. Among CD45^+^ immune cells, the increase in CD44-expressing cells by BBP treatment occurred mainly within the pDC subset but not within cDCs (Figure 3C,D) or monocytes (Supplementary Figure S4). In contrast, ICAM-1 expression was not altered in the infiltrated CD45^+^ immune cells, including either the pDC or cDC subset, or in the CD45^−^ nonimmune cells in the BBP-treated group (Supplementary Figure S5). The results suggest that CD44 expression in pDCs may be involved in lesion survival in this surgically induced murine model of endometriosis.

### 3.4. Blocking Interactions with CD44 Inhibits Endometriotic Lesion Development and CD44^+^ pDC Infiltration into Endometriotic Lesions

**Figure 4:**
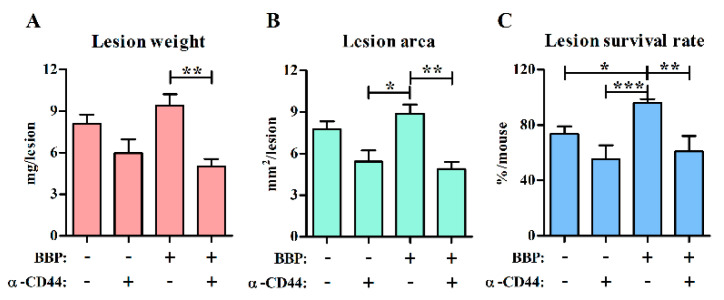
Effect of CD44 blocking on lesion growth. Lesions were harvested after 4 weeks from the control and BBP-treated (1.5 mg BBP/kg BW/day) mice. Each mouse was treated with blocking antibody against CD44 on one side (+) and isotype control antibody on the other side (–) at the implantation sites on the day of surgery. (**A**−**C**) Lesion weight (**A**), lesion area (**B**), and lesion survival rate (**C**) per mouse are shown as the mean ± SEM for each group (*n* = 9–13 mice/group from four independent experiments). * *p* < 0.05, ** *p* < 0.01 and *** *p* < 0.001 by one-way ANOVA followed by Tukey’s multiple comparison test.

To explore whether CD44 is involved in BBP-mediated lesion survival, a blocking monoclonal antibody against CD44 was intradermally injected under the sutured endometrial tissue (Supplementary Figure S2A) to block interactions between CD44 and its ligand during the early stage of lesion development. Its corresponding isotype control was injected on another side within the same mouse for decreasing individual variation. To clarify that the injected antibody remains locally near sutured tissue, a mouse was intradermally injected with PE-conjugated antibody into the peritoneal wall, and the fluorescence was tracked by the in vivo imaging system. The result showed that the injected PE-conjugated antibody remained locally at least within 120 min after injection (Supplementary Figure S2B). The gradual decrease in fluorescence may be due to the fluorescence decrease of the labeled antibody. If the antibody was injected into the peritoneal cavity, the fluorescence intensity would quickly disappear. These data indicate that the injected antibody may remain locally at the site of the endometrial lesion to modulate the development of endometriosis at least during the early stage in the model. The results showed that the blocking treatment for CD44 not only tended to inhibit lesion growth in the control group but also significantly decreased the weight, area, and survival rates of endometriotic lesions in the BBP-treated group (Figure 4).

**Figure 5:**
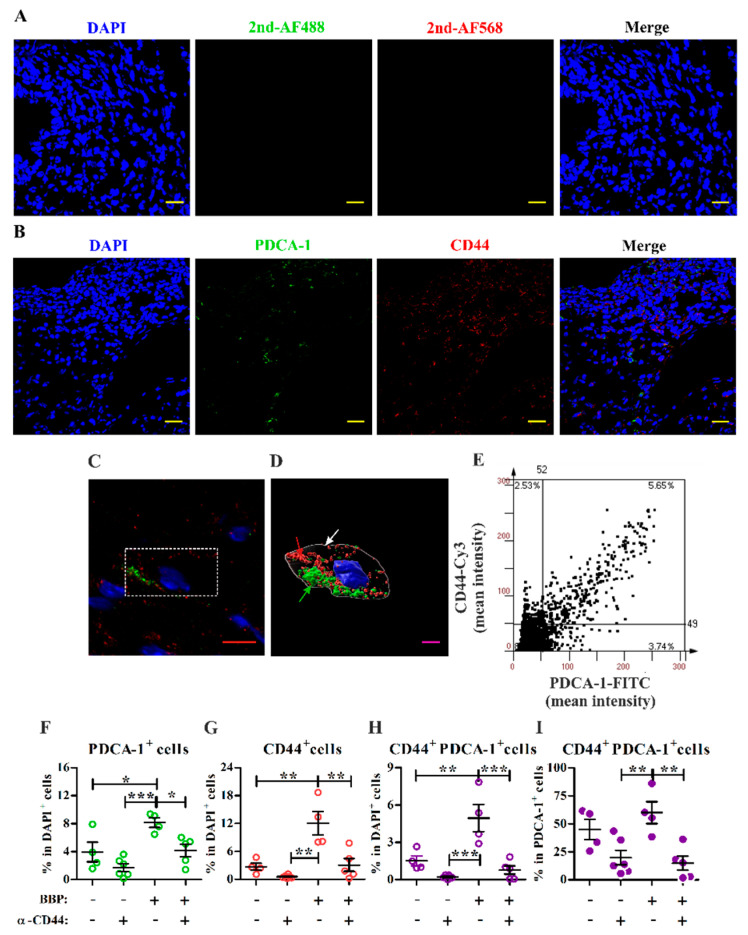
Identification of CD44-expressing pDCs and the effect of a CD44 blocking antibody on the pDC subset in endometriotic lesions. Representative immunofluorescence images of endometriotic lesion sections from one BBP-treated mouse. Lesion sections were stained with secondary antibodies alone (**A**) and with primary antibodies (PDCA-1 and CD44) and secondary antibodies (**B**), showing PDCA-1^+^, CD44^+^, and CD44^+^PDCA-1^+^ cells. Scale bars = 20 µm. (**C**) The immunofluorescence image of one representative CD44^+^PDCA-1^+^ cell, as shown in the dashed white box. Scale bar = 5 µm. (**D**) The image of the representative cell shown in (**C**) was captured by confocal z-stack scanning and was remodeled as a three-dimensional structure in a basic way showing PDCA-1 (green arrow) and CD44 (red arrow) expression with respect to the putative cell membrane (white arrow). Scale bar = 3 µm. (**E**) A representative dot plot is shown for quantification of PDCA-1^+^, CD44^+^, and CD44^+^PDCA-1^+^ cells from immunofluorescence images of an intact BBP-treated endometriotic lesion. The *x*-axis shows the mean fluorescence intensity of PDCA-1, and the *y*-axis indicates CD44 fluorescence. (**F**–**I**) The frequencies of PDCA-1^+^ (**F**), CD44^+^ (**G**), and CD44^+^PDCA-1^+^ (**H**) cells among DAPI^+^ cells and the frequency of CD44^+^PDCA-1^+^ cells among PDCA-1^+^ cells (**I**) from control and BBP-treated mice were quantified for an entire field from each lesion section. The lesions were collected from each mouse locally treated with blocking monoclonal antibody against CD44 on one side (+) and with an isotype control antibody on the other side (−) at the implantation sites. Each data point in (**F**–**I**) represents one section from one endometriotic lesion, and only one endometriotic lesion was quantified per mouse. The black horizontal lines and error bars represent the mean and SEM for each group, respectively (*n* = 4–6 mice/group from two independent experiments). * *p* < 0.05, ** *p* < 0.01, and *** *p* < 0.001 by one-way ANOVA followed by Tukey’s multiple comparison test.

Next, double immunofluorescence staining for CD44 and PDCA-1, a representative marker for pDCs, was used to analyze the effect of CD44 blocking treatment on the frequency of pDCs in lesions. Compared with a representative lesion section stained with secondary antibodies and DAPI to indicate background staining, we observed PDCA-1^+^, CD44^+^, and CD44^+^PDCA-1^+^ cells in the lesion sections (Figure 5A,B). One representative cell (dashed white box) from a BBP-treated lesion was selected (Figure 5C), and its putative membrane was remodeled based on CD44 and PDCA-1 expression (Figure 5D). The three-dimensional structure of the cell showed the typical morphology of a pDC, with an oval shape, smooth surface, and eccentric nucleus [32]. Immunofluorescence quantitative analysis (Figure 5E) showed that BBP exposure increased the percentages of PDCA-1^+^ cells, CD44^+^ cells, and CD44^+^PDCA-1^+^ cells (Figure 5F–H) in endometriotic lesions. As we expected, CD44 blocking at the early stage significantly decreased the frequencies of PDCA-1^+^, CD44^+^, and CD44^+^PDCA-1^+^ cells among all DAPI^+^ cells in BBP-treated lesions. The proportion of CD44^+^ cells among all PDCA-1^+^ cells was significantly reduced after CD44 blocking in the BBP group (Figure 5I). Taken together, these results suggest that infiltrated pDCs may promote BBP-mediated lesion survival in a CD44-dependent manner.

### 3.5. Chronic Exposure to Low-Dose BBP Alters the Estrous Cycle in Mice

**Figure 6:**
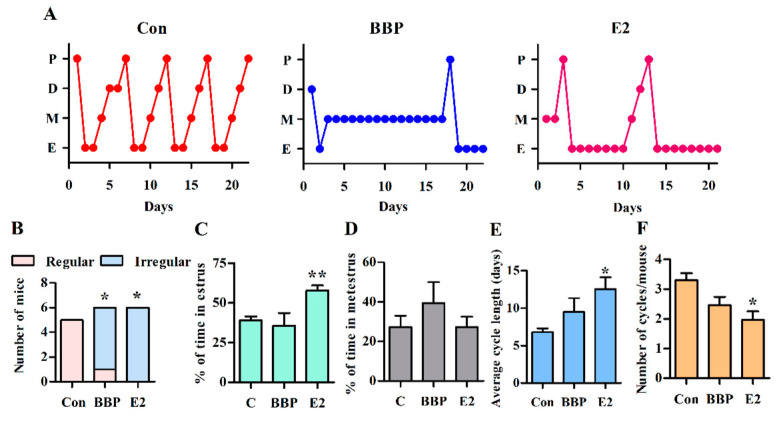
BBP disrupts the estrous cycle. (**A**) Estrous cycles for representative control (Con), BBP-treated (1.5 mg/kg BW/day), and E2-treated (50 µg/kg BW/day) mice over 22 days of treatment. D, diestrus; E, estrus; M, metestrus; P, proestrus. (**B**) The number of mice with regular and irregular cycles in each group (Mid-P exact test, * *p* < 0.05). (**C**–**F**) The percentage of time spent in the estrus (**C**) and metestrus (**D**), average cycle length (**E**), and the number of cycles per mouse (**F**). The percentage of time was determined based on the days in the full treatment days. Data are pooled from two independent experiments (control, *n* = 5; BBP, *n* = 6; E2, *n* = 6). Results are shown as the mean ± SEM. * *p* < 0.05; ** *p* < 0.01 vs. control using the Mann–Whitney U test.

Previous studies have shown that BBP has low estrogenic activity [17,18], so we analyzed whether BBP exposure consisting of the 3× TDI daily dose affects the estrous cycle in mice. In a small set of experiments, all control mice showed regular cycles (Supplementary Figure S6); however, most of the BBP-exposed mice (five of six mice) displayed irregular cycles, i.e., >50% of total cycle time was spent in one stage, with the exception of one mouse with a regular cycle (Figure 6A,B). In contrast to E2-exposed mice, which have a prolonged estrus stage, BBP-exposed mice tended to remain in metestrus (Figure 6A,C,D), which resulted in a prolonged cycle length and a decreased cycle number over the 22-days exposure (Figure 6E,F). The changes in the estrus cycle mediated by BBP exposure are similar to E2 but some parameters did not reach statistical significance may be due to the small number of mice examined. Taken together, these results indicate that chronic exposure to low-dose BBP may affect hormone regulation as well as innate responses in the context of endometriosis.

## 4. Discussion

BBP, a ubiquitous environmental pollutant, can interfere with and disrupt endocrine systems due to its low estrogenic activity. Our study showed that BBP exposure at the human TDI dose enhances the survival rate of endometriotic lesions. However, the relative frequencies of infiltrating immune cell subsets were not altered by BBP exposure, except for the frequency of CD44^+^ cells among the pDC subset. Immunofluorescence analysis of endometriotic lesions showed that blocking the CD44 interaction significantly reduced pDC infiltration as well as CD44 expression among pDCs in the BBP-treated mice, suggesting the involvement of CD44-expressing pDCs in lesion development. This study suggests that BBP exposure may affect the survival rate of endometriotic lesions and that this effect is associated with elevated expression of CD44 on pDCs in the context of endometriosis.

CD44 is expressed by various types of immune cells and nonimmune cells and is involved in multiple biological functions such as adhesion, migration, hematopoiesis, lymphocyte activation, homing, and extravasation [33]. In the context of endometriosis, CD44 expressed by ectopic endometrial cells is linked to aberrant cell adhesion. Previous studies have demonstrated that CD44–hyaluronic acid binding is associated with the peritoneal adherence of ovarian carcinoma cells in vitro [34,35]. Using endometriosis murine models, Tsai et al. have shown that decoy receptor 3 enhances the adhesion and migration of ectopic endometrial cells by inducing the expression of CD44 and ICAM-1 in the endometrium [31]. In contrast, CD44 is also expressed by DCs and participates in their trans endothelial migration [36] and ability to stimulate T-cells [37]. However, to the best of our knowledge, it remains to be determined whether pDCs express CD44 and what their function in the context of this disease is. In the present study, we observed that approximately one-third of infiltrated pDCs expressed CD44 in the lesions in the experimental setting, whereas BBP exposure increased CD44 expression on infiltrated pDCs or enhanced the infiltration of CD44^+^ pDCs into lesions. Blocking CD44 interactions at the beginning of endometrial tissue attachment significantly decreased lesion size, weight, and pDC frequency in the BBP-treated group. These changes mediated by CD44 blocking are in addition to the BBP effect on lesion growth, suggesting multiple functions of CD44 in the pathogenesis of endometriosis.

Both cDCs and pDCs are involved in the pathogenesis of endometriosis in humans and in mice. The density of CD1a^+^ (immature) DCs is increased during the proliferative phase in the basal layer of the endometrium, and the density of CD83^+^ (mature) DCs is decreased across all stages of the menstrual cycle in women with endometriosis [38]. Pencovich et al. demonstrated that DC depletion using diphtheria toxin reduces lesion size, suggesting a role for DCs in the development of endometriotic lesions [39]. Our previous study showed that local IL-10 activity promotes the development of endometrial lesions in a surgically induced murine model and that the major IL-10-secreting immune cells in endometriotic lesions are pDCs [6,7]. We further demonstrated that the IL-10–IL-10 receptor axis promotes angiogenesis in vitro and in vivo and promotes endometriosis pathogenesis during the early stage of disease [6]. In support of these previous studies, BBP exposure may enhance the expression of CD44 on infiltrated pDCs and is likely to contribute to the survival of endometrial tissue in the peritoneal cavity. However, the mechanism by which the CD44–pDC axis participates in lesion development awaits further investigations.

In our previous study, we showed that 4-nonylphenol, an EED, alters the estrous cycle in mice [28]. Another EED, bisphenol A, at a dose of 1.2 mg/kg BW/day, also alters estrous cyclicity in Sprague–Dawley female rats [40]. In this study, BBP exposure showed a similar effect, resulting in an irregular estrous cycle in female mice, including a tendency toward a shortened cycle length and thus an increase in the number of cycles. In contrast to treatment with E2, the effect of BBP on cycle number and length did not reach statistical significance, possibly due to the low affinity of BBP for ERs as compared with E2 (<0.001) [14] or the small number of mice used in this set of experiments. However, although BBP has low estrogenic activity, similar to 4-nonylphenol, exposure to BBP resulted in mice that stayed in metestrus, whereas mice treated with 4-nonylphenol or E2 remain in estrus for longer periods of time [28]. This result suggests that the BBP-mediated mechanism that underlies an irregular estrous cycle is likely to be different from that in response to 4-nonylphenol exposure or E2 treatment. The effect of long-term exposure to BBP at environmental doses on estrogenic regulation still needs to be investigated in depth.

The main limitation of the surgically induced endometriosis model used here is the inability to replicate the natural adhesion mechanism because the endometrial fragments are sewn to the peritoneal cavity. However, the peritoneal injection model [41,42] was not suitable for the aim of this study, which required that we avoid treating the endometrial tissue with exogenous E2 in vivo and removing the ovary prior to surgery or intraperitoneal injection into autologous mice or syngeneic recipient mice, respectively. A better endometriosis model is needed for dissecting the causal relationship between EED exposure and endometriosis development.

## 5. Conclusions

In conclusion, we have demonstrated that chronic exposure to low-dose BBP promotes lesion survival in a surgically induced endometriosis murine model. The BBP-mediated lesion survival may be associated with local CD44-expressing pDCs in the endometriotic lesions; however, a detailed mechanism requires further investigation. In addition, BBP exposure also disrupted estrous cyclicity in the mice. This study suggests a potential link between EED exposure and endometriosis development in women.

## Data Availability

The data presented in this study are available on request from the corresponding author. The participants gave permission to learn and handle their personal and research-generated data only to researchers involved in this study. The data are not publicly available.

## Supplementary Materials

The following are available online at https://www.mdpi.com/article/10.3390/ijerph18073640/s1, Figure S1. Schematic depiction of the experimental protocol, Figure S2. In vivo imaging of mAb distribution in the endometriosis-like model, Figure S3. Effects of BBP on monocyte and macrophage populations in endometriotic lesions, Figure S4. BBP exposure did not enhance CD44 expression in monocytes, Figure S5. BBP exposure did not enhance ICAM-1 expression in pDCs or in cDCs, Figure S6. Cytological evaluation of vaginal smears to identify estrous cycle stages.
