# Supplementary material for: n-Butyl Benzyl Phthalate Exposure Promotes Lesion Survival in a Murine Endometriosis Model
Source: Int J Environ Res Public Health. 2021 Mar 31;18(7):3640. doi: 10.3390/ijerph18073640 (PMC8036315; doi:10.3390/ijerph18073640)
Supplement: Supplementary file 1 [file ijerph-18-03640-s001.pdf]

_n_-Butyl benzyl phthalate exposure promotes lesion survival in a murine endometriosis model

Pooja Sharma[1], Jo-Yu Lynn Lee[1], Eing-Mei Tsai[1,2], Yu Chang[3†], Jau-Ling Suen[1,4,5†,*]

Supplementary materials

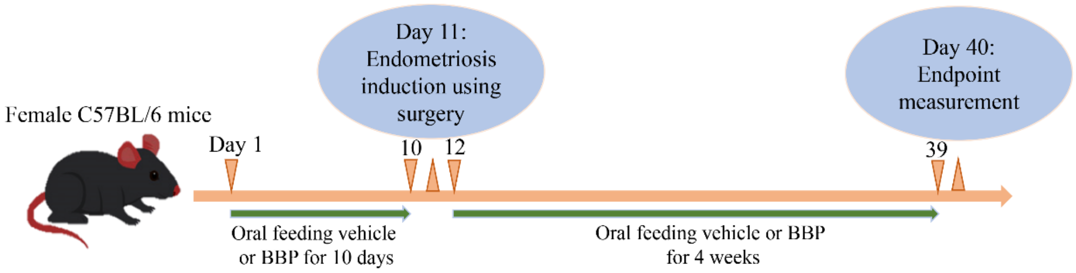

Supplementary Figure S1. Schematic depiction of the experimental protocol. Female C57BL/6 mice were orally fed with either vehicle alone or BBP (0.5 or 1.5 mg/kg BW/day) daily. After 10 days of oral feeding, the mice underwent surgery to suture endometrial tissue to the abdominal wall on day 11. On the next day, the oral feeding of vehicle or BBP was resumed for an additional 4 weeks. The lesions were harvested for further analysis on day 40.

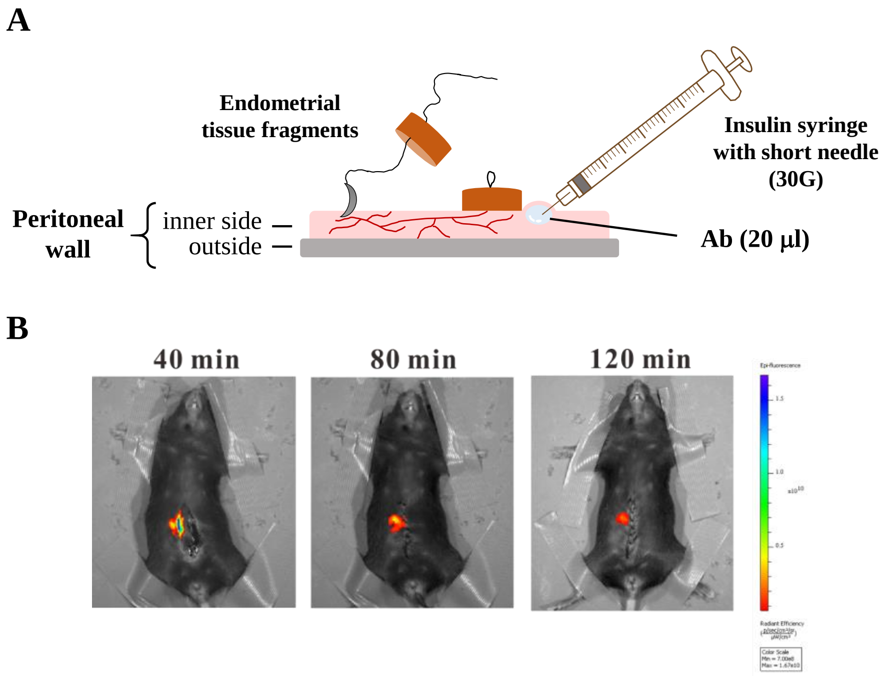

Supplementary Figure S2. *In vivo* imaging of mAb distribution in the endometriosis-like model. (**A**) C57BL/6 female mouse was surgically sutured the endometrial lesion as described in the materials and method section. PE-conjugated mAb (20 μg/ml, 20 μl/lesion) was subcutaneously injected into the inner side of peritoneal wall under or close to the transplanted tissue on the right side and PBS on the left side. (**B**) The mouse was performed the *in vivo* bio-fluorescence imaging using the IVIS spectrum imaging system (Caliper Life Sciences) at 40, 80 and 120 minutes after mAb injection. mAb: monoclonal antibody.

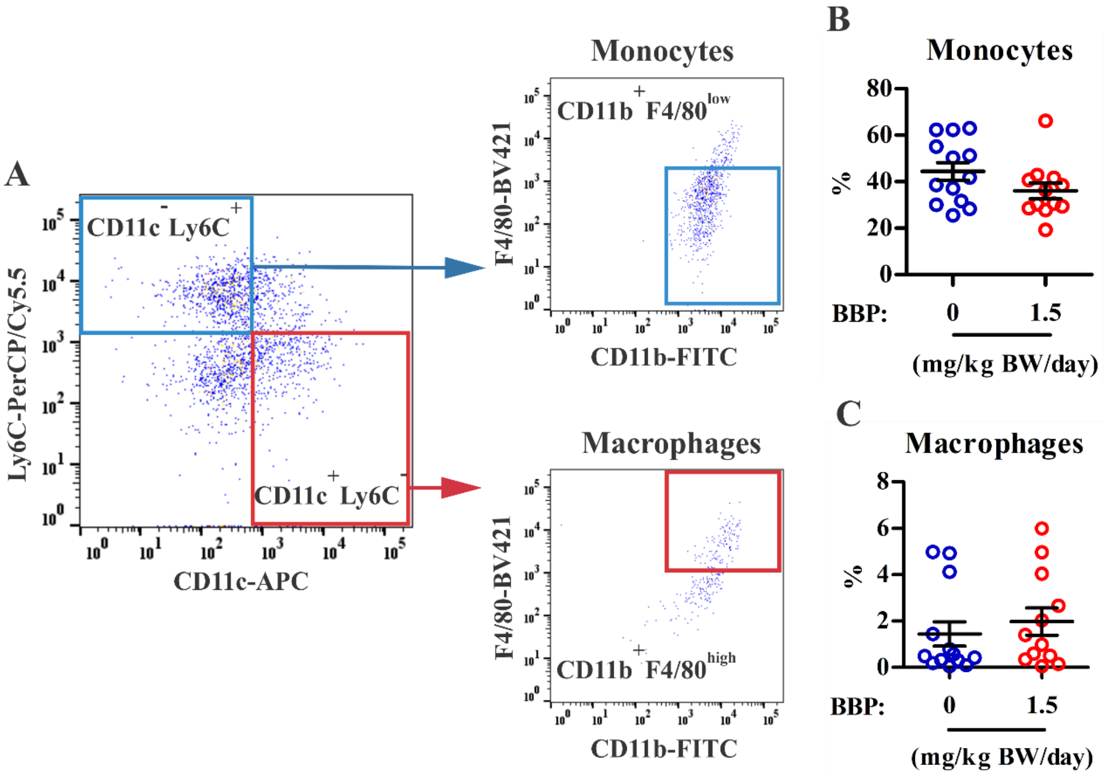

Supplementary Figure S3. Effects of BBP on monocyte and macrophage populations in endometriotic lesions. Single cells from lesions were analyzed using flow cytometry for monocytes and macrophage populations. (**A**) Representative dot plots show the gating strategy for monocytes and macrophages in live CD45$^+$ cells from BBP-treated mice (1.5 mg/kg BW/day). (**B**,**C**) Percentages of monocytes (**B**) and macrophages (**C**) in pooled endometriotic lesions from each mouse. Each data point represents one mouse. The data in (**B**,**C**) are from four independent experiments, with a total of n = 13 mice in the control group and *n* = 12 mice in the BBP group. Each mark in (**B**,**C**) represents data from one mouse. The black horizontal lines and error bars in (**B**,**C**) represent the mean and SEM, respectively, for each group.

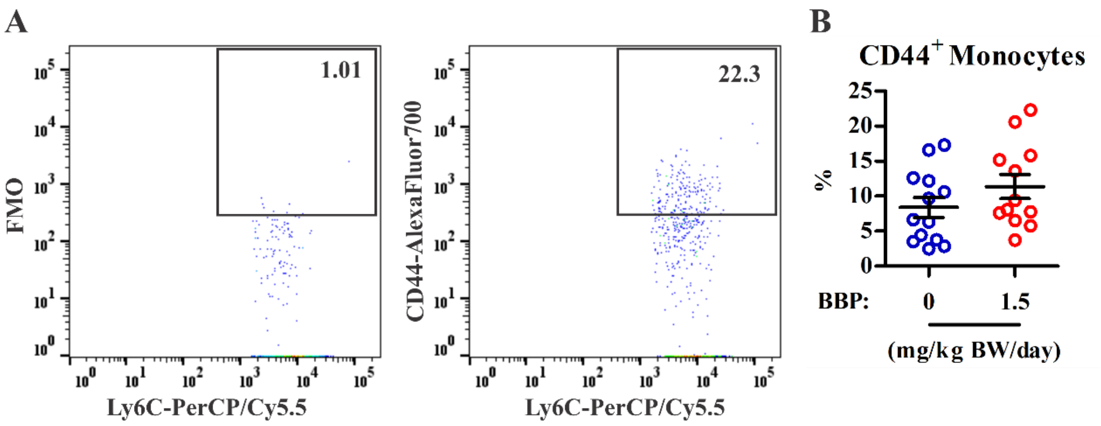

Supplementary Figure S4. BBP exposure did not enhance CD44 expression in monocytes. Lesions were harvested and pooled from each single mouse for analysis of CD44 expression among immune cells. (**A**) Representative dot plots are shown for CD44 expression in monocytes from BBP-treated mice (1.5 mg/kg BW/day). The $CD44^+$ cells were gated according to the fluorescence minus one (FMO) control. (**B**) Percentages of $CD44^+$ cells in monocytes from control and BBP groups (control, n = 13 mice; 1.5 mg BBP, n = 12 mice). The data shown are from four independent experiments. Each data point in (**B**) represents data from one mouse. The black horizontal lines and error bars represent the mean and SEM, respectively, for each group.

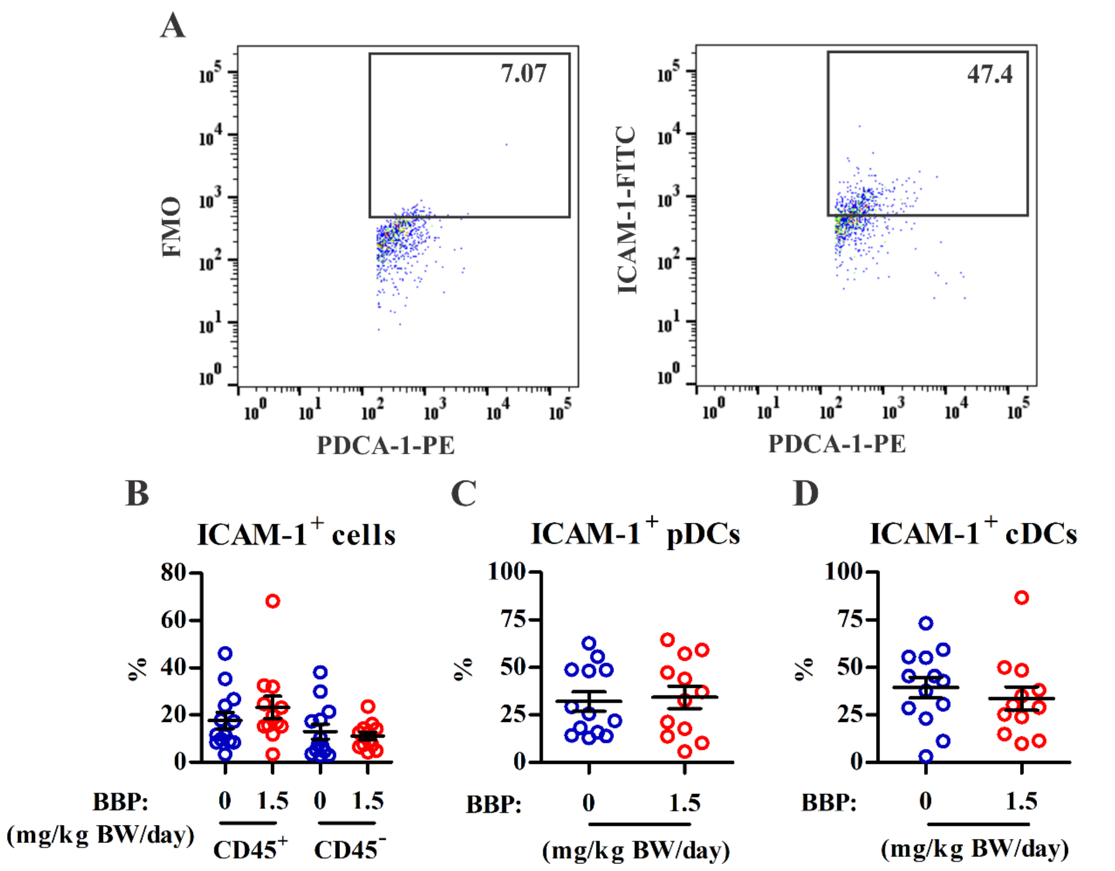

Supplementary Figure S5. BBP exposure did not enhance ICAM-1 expression in pDCs or in cDCs. Lesions were harvested and pooled from each single mouse for analysis of ICAM-1 expression among immune cells. (**A**) Representative dot plots are shown for ICAM-1 expression in pDCs from BBP-treated mice (1.5 mg/kg BW/day). The ICAM-1$^+$ cells were gated according to the fluorescence minus one (FMO) control. (**B**) Percentages of ICAM-1$^+$ cells among CD45$^+$ immune and CD45$^-$ non-immune cells from control and BBP-treated mice. (**C**,**D**) Percentages of ICAM-1$^+$ cells in pDCs (**C**) and cDCs (**D**) from control and BBP-treated lesions (control, *n* = 13 mice; BBP, *n* = 12 mice). The data in (**B–D**) are from four independent experiments. Each data point in (**B–D**) represents data from one mouse. The black horizontal lines and error bars represent the mean and SEM, respectively, for each group.

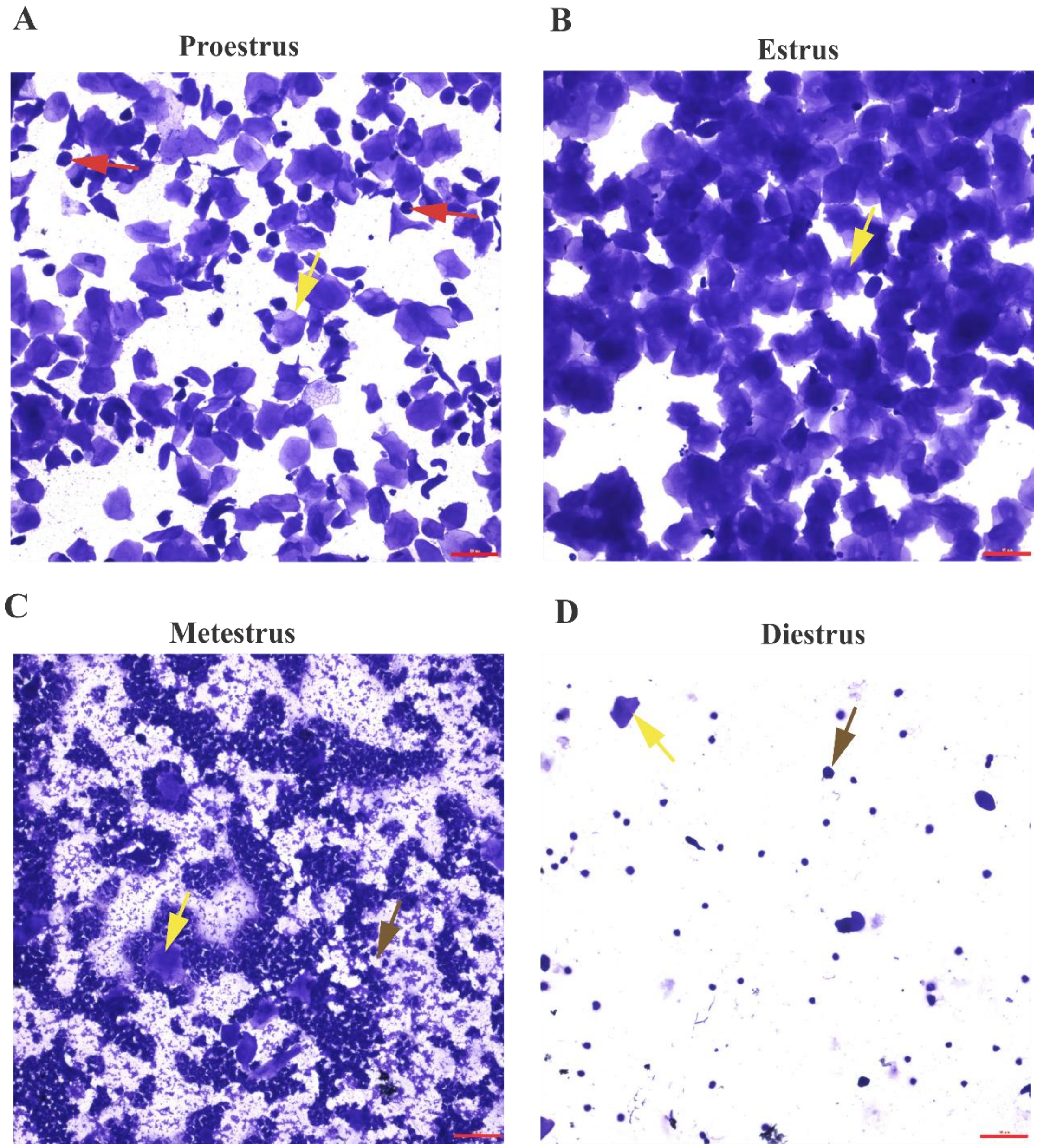

Supplementary Figure S6. Cytological evaluation of vaginal smears to identify estrous cycle stages. (**A–D**) Representative images of four estrous stages from one control mouse, showing proestrus (**A**), estrus (**B**), metestrus (**C**), and diestrus (**D**). Three different type of cells were observed in the vaginal smear samples, nucleated epithelial cells (red arrows), anucleated cornified epithelial cells (yellow arrows), and neutrophils (brown arrows). The ratios of these cell types present in each smear were used to identify the four estrous cycle stages as described in the Materials and Methods. Scale bars = 50 μm.
